# Extending Postpartum Medicaid Beyond 60 Days Improves Care Access and Uncovers Unmet Needs in a Texas Medicaid Health Maintenance Organization

**DOI:** 10.3389/fpubh.2022.841832

**Published:** 2022-05-03

**Authors:** Xiao Wang, Yolande Mfondoum Pengetnze, Emily Eckert, Graham Keever, Vikas Chowdhry

**Affiliations:** ^1^Parkland Center for Clinical Innovation (PCCI), Dallas, TX, United States; ^2^American College of Obstetricians and Gynecologists (ACOG), Washington, DC, United States; ^3^Parkland Community Health Plan (PCHP), Dallas, TX, United States

**Keywords:** postpartum coverage, Medicaid extension, health services utilization, maternal health, rapid repeat pregnancy

## Abstract

Under longstanding federal law, pregnancy-related Medicaid coverage is only guaranteed through 60-days postpartum, at which point many women become uninsured. Barriers to care, including lack of insurance, contribute to maternal mortality and morbidity. Leveraging the Families First Coronavirus Response Act, a federal law requiring that states provide continuous coverage to Medicaid enrollees during the COVID-19 pandemic as a condition of receiving enhanced federal financial support, we examine whether postpartum women seek additional care, and what types of care they use, with extended coverage. We analyze claims from the Parkland Community Health Plan (a Texas Medicaid Health Maintenance Organization) before and after implementation of the pandemic-related Medicaid extension. We find that after implementation of the coverage extension, women used twice as many postpartum services, 2 × to 10 × as many preventive, contraceptive, and mental/behavioral health services, and 37% fewer services related to short interval pregnancies within the first-year postpartum. Our findings provide timely insights for state legislators, Medicaid agencies, and members of Congress working to improve maternal health outcomes. We add empirical evidence to support broad extension of Medicaid coverage throughout the first-year postpartum.

## Introduction

Under longstanding federal law, individuals eligible for Medicaid on the basis of pregnancy are only guaranteed 60 days of postpartum coverage (1). After this arbitrary cliff, many women become uninsured (2, 3). Women's health experts, including the American College of Obstetricians and Gynecologists (ACOG) (4), maintain that inadequate access to health insurance during the full year postpartum contributes to preventable maternal mortality and morbidity.

Some state legislatures and Medicaid programs across the country are considering extending the postpartum coverage period beyond 60 days to improve maternal health outcomes. Most states opted to pursue iterations of this policy via Section 1115 waiver authority. Then, in March of 2021, the American Rescue Plan Act (ARPA) created a new, streamlined pathway for states to extend postpartum coverage via a Medicaid state plan amendment (SPA) (5). Weeks later, the Biden administration issued its first approval of a Section 1115 waiver from the state of Illinois to extend coverage (6).

Since the passage of ARPA and the approval of the Illinois waiver, state activity to adopt this policy has increased (7). As a result of this momentum, the United States Congress is considering building on the ARPA SPA by requiring states to extend coverage for pregnant women who rely on Medicaid for pregnancy-related care to 12 months after the end of pregnancy as part of the Build Back Better agenda (8). Questions remain, however, regarding whether individuals will utilize additional health care services and experience improved outcomes with extended postpartum coverage.

A provision in the Families First Coronavirus Response Act (FFCRA) of 2020 provides a unique opportunity to study the potential impact of extending the Medicaid postpartum coverage period. By requiring that state Medicaid programs provide continuous coverage to enrollees through the end of the COVID-19 public health emergency (PHE), the FFCRA functionally extends coverage for pregnant women beyond 60 days postpartum (9, 10).

While decades of health services research demonstrate that broadening access to health insurance, including through Medicaid, improves insurance coverage and maternal health outcomes, no study has used claims data to examine the impact of the FFCRA's continuous coverage provision on postpartum utilization in real time (11–13). Thus, the objectives of this study were to determine whether postpartum women use additional health care services with extended Medicaid coverage, what types of services they use, and what diagnoses are associated with their postpartum health services utilization. These findings are timely and help inform the ongoing debates in state legislatures and Congress about the benefits of extending postpartum coverage under Medicaid.

## Methods

We conducted a retrospective cohort study of postpartum women using Medicaid claims data from the Parkland Community Health Plan (PCHP). Of note, pharmacy claims were not included in these analyses. We analyzed all singleton deliveries between 06/01/2019 and 12/31/2020 among women aged 14–48 years old. Two postpartum cohorts were identified: a Pre-FFCRA cohort of women who delivered before 01/17/2020 and were, therefore, not impacted by the coverage extension, and a Post-FFCRA cohort of women who delivered on or after 01/17/2020 and were eligible for the coverage extension (which went into effect 03/18/2020). Women covered by Traditional Medicaid, the Children's Health Insurance Program (CHIP) unborn child option, and those with invalid eligibility records were excluded (see Supplementary Figure 1). A cohort of Medicaid-insured children age 2–17 was identified as an external comparison group to control for secular trends and pandemic-related effects on utilization.

In a secondary analysis, the Pre-FFCRA cohort was further divided into women who experienced a coverage termination at 60 days postpartum (i.e., without subsequent coverage) and women who remained eligible for Medicaid after 60 days postpartum under a different eligibility category (i.e., with subsequent coverage) to further characterize the impact of extended postpartum coverage pre-pandemic. Eligibility categories for subsequent Medicaid coverage included pregnancy-related Medicaid (for new pregnancies), Temporary Assistance for Needy Families (TANF), and Medicaid for children under age 19 for the teenage mothers.

Primary outcomes were health services utilization, including postpartum and all-cause outpatient services, mental/behavioral health (MBH) and substance use disorder (SUD) services, preventive and problem-related services, and emergency department (ED) and inpatient services (14). All COVID-related diagnoses were removed from the analyses to avoid any skewness of Post-FFCRA data due to COVID-related utilization. Outcomes were measured in the first-year postpartum across the following time intervals: 7–90-days; 91–182-days (i.e., 3–6-months); and 183–365-days (i.e., 6–12-months). The 90-days cutoff was used to reflect that, under federal law, pregnancy-related Medicaid eligibility continues through the end of the month in which the 60-days postpartum period ends (1). Coverage for pregnant women, therefore, might continue for up to 90-days postpartum when the end of the 60-days postpartum coverage period overlaps with the beginning of a calendar month. We, therefore, used the 90-days upper-bound cutoff for all women (rather than actual end-of-coverage date) as a stringent benchmark and to facilitate comparison with the Post-FFCRA cohort. Sensitivity analyses using actual end-of-coverage date yielded similar findings (data not shown). Utilization was calculated for each cohort either by dividing the number of members with a specific type of claim by the cohort size (percent cohort) or by dividing the number of claims of a specific type by the cohort size (visit rates per 100 members).

The primary analysis was to measure between-cohort differences in health services utilization, comparing Pre-FFCRA vs. Post-FFCRA cohorts. Adjusted analyses were not performed because baseline characteristics were comparable between cohorts. For outpatient services claims, primary diagnoses were identified and categorized as postpartum, general exam, pregnancy-related, or contraceptive services. Furthermore, diagnoses and places of service were used to categorize claims as MBH/SUD, preventive, problem-related, or acute care (ED and inpatient) services.

The secondary analysis was a three-way comparison of health services utilization among Pre-FFCRA women without subsequent coverage vs. Pre-FFCRA women with subsequent coverage vs. Post-FFCRA women. Results of the secondary analysis are reported in the Supplementary Materials, along with additional methodologic details, including a cohort creation diagram, variables definitions, and a comparison of baseline cohort characteristics.

## Results

Included in the analysis were 3,465 Pre-FFCRA and 5,411 Post-FFCRA deliveries. Socio-demographic characteristics were comparable between cohorts (Supplementary Table 1).

### Postpartum and Outpatient Services Utilization

**Figure 1:**
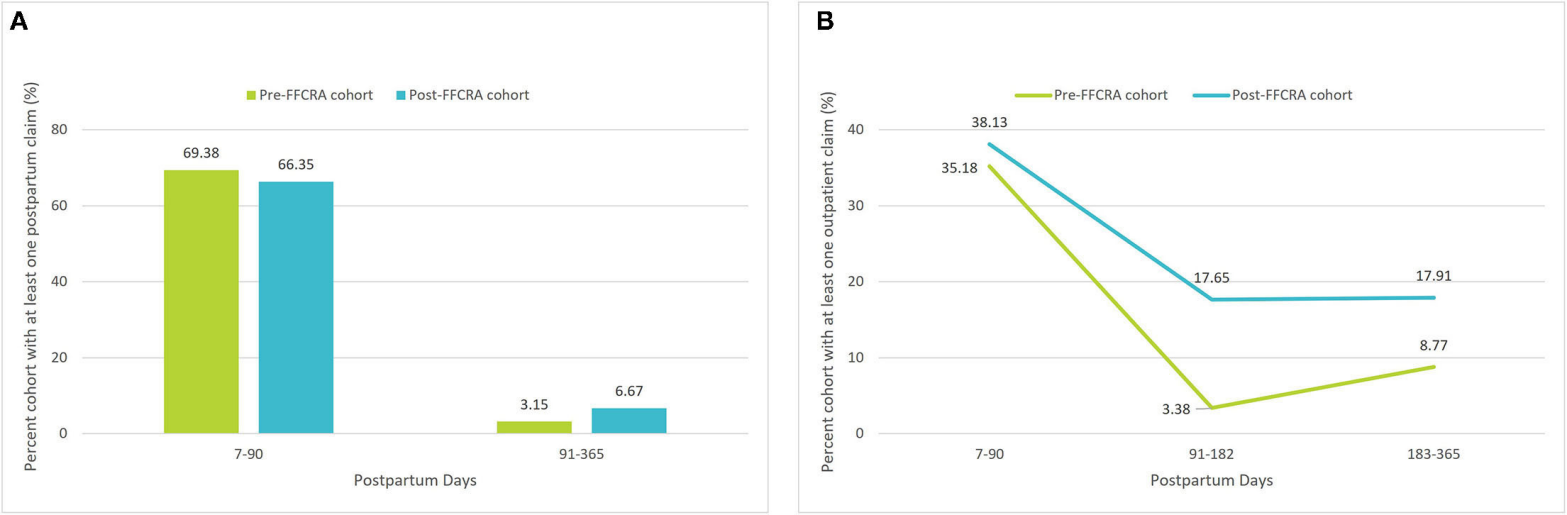
**(A)** Postpartum outpatient services utilization, before vs. after FFCRA implementation. **(B)** All-cause outpatient services utilization, before vs. after FFCRA implementation.

Figure 1A depicts changes in postpartum services utilization (as defined by HEDIS criteria) and Figure 1B depicts trends in overall outpatient services utilization, comparing Post-FFCRA vs. Pre-FFCRA cohorts. Within 90 days of delivery, postpartum services utilization was comparable Post-FFCRA vs. Pre-FFCRA (Figure 1A). After 90 days postpartum, however, Post-FFCRA utilization was 2-fold higher than Pre-FFCRA utilization (6.7 vs. 3.2%, respectively; Figure 1A). The same patterns were observed when examining all outpatient services utilization. Although overall outpatient services utilization decreased after 90-days postpartum, Post-FFCRA utilization was 2–5-fold higher than Pre-FFCRA utilization through the end of the first-year postpartum, with 17.7% of Post-FFCRA women receiving outpatient care between 91- and 182-days and 17.9% between 183- and 365-days postpartum, vs. 3.4 and 8.8% for Pre-FFCRA women, respectively. Further analyses revealed that, in the Pre-FFCRA cohort, all outpatient utilization after 90-days postpartum occurred among women with subsequent coverage while no utilization was recorded after 90-days postpartum for women without subsequent coverage (See Methods and Supplementary Figure 3 for details).

### Reasons for and Types of Services Utilization

**Figure 2:**
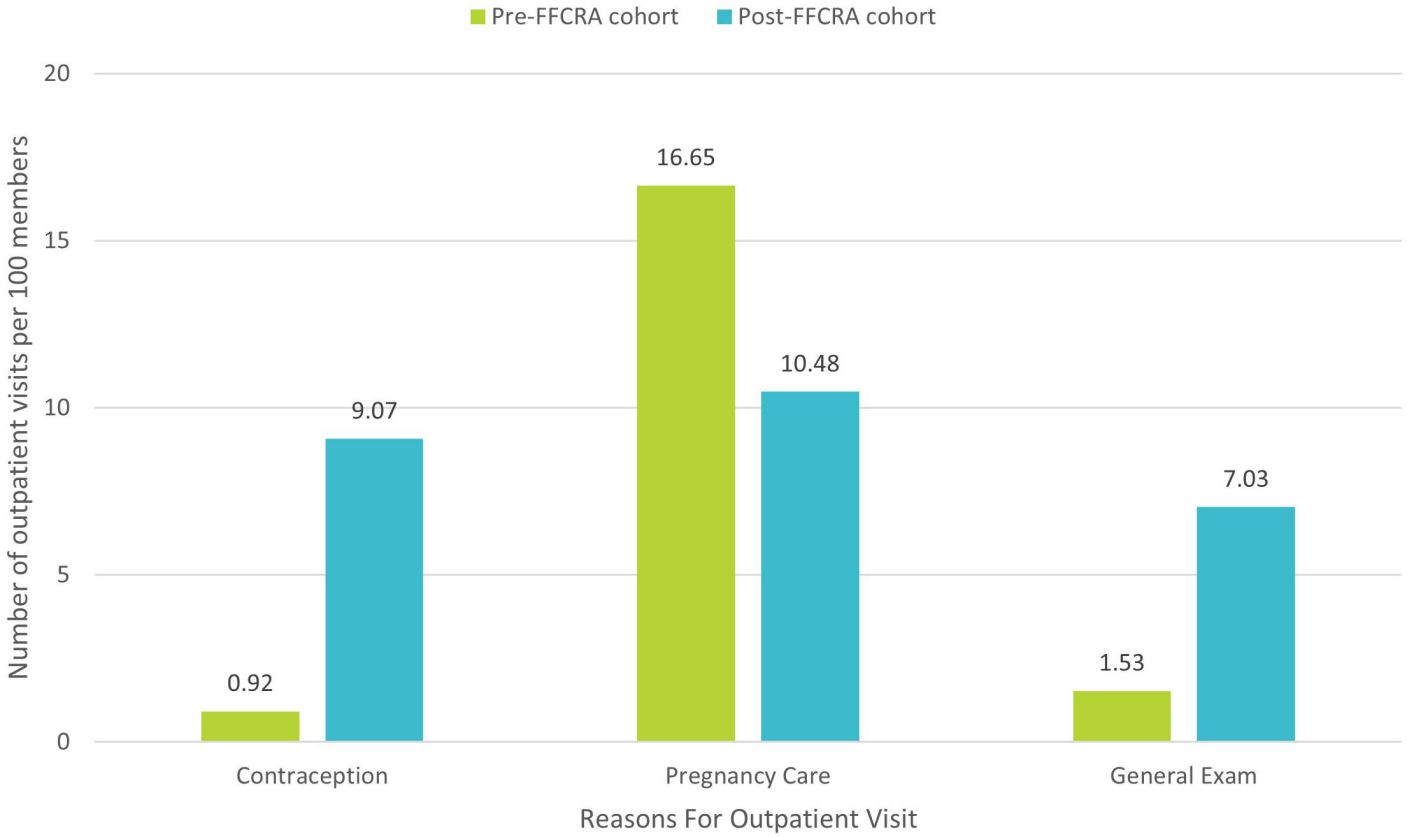
Reasons for visits among the top 30 primary diagnoses driving outpatient utilization between 91- And 365-days postpartum (Excluding COVID-19-related diagnoses; details in Supplementary Materials).

**Figure 3:**
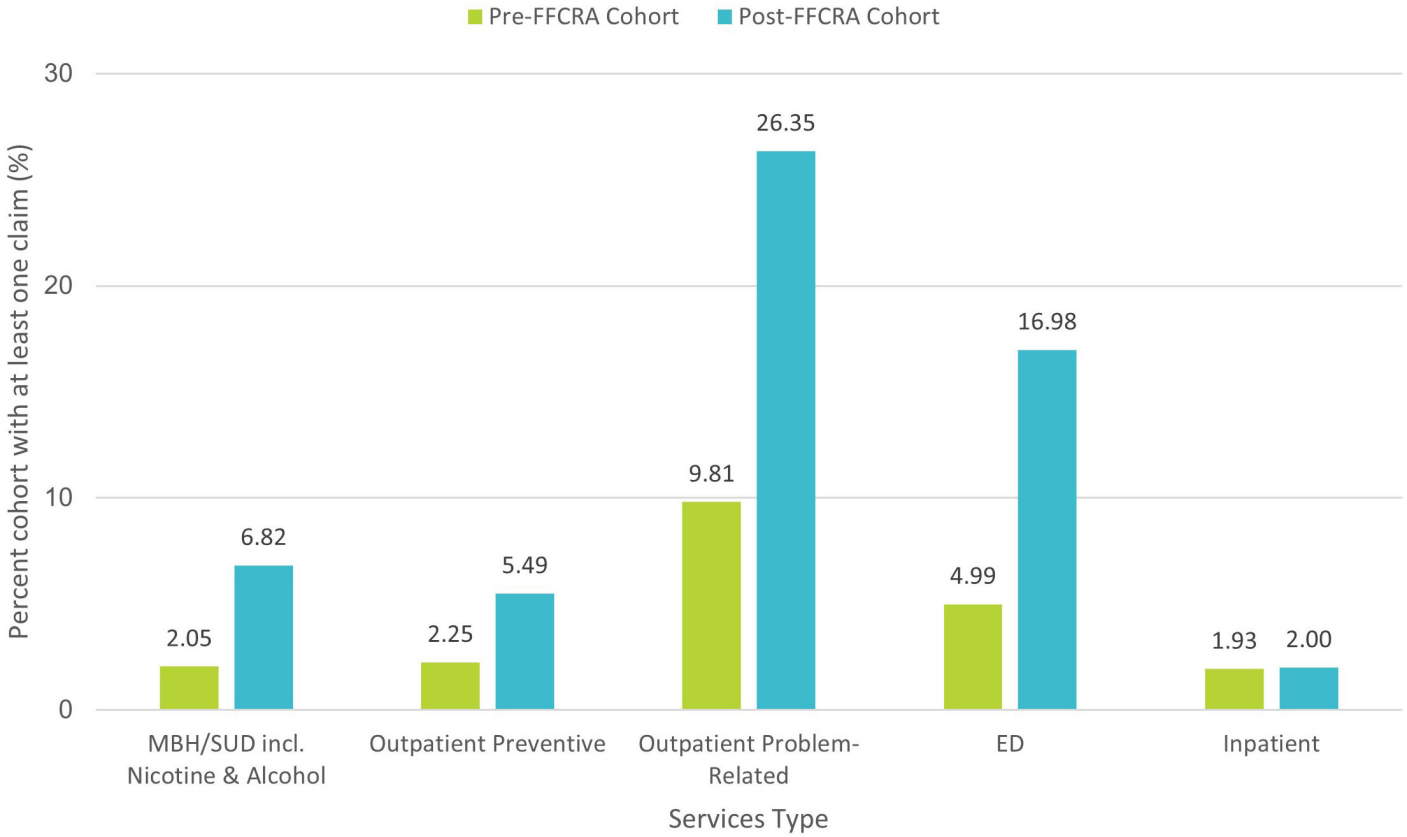
Services type—Mental/Behavioral Health (MBH), Substance Use Disorder (SUD) and acute vs. preventive health services utilization between 91- and 365-days postpartum, before vs. after FFCRA implementation.

In further analysis of the reasons for services utilization, Post-FFCRA women used 10 × as many contraceptive services and 37% fewer services for subsequent pregnancies within the first-year postpartum (37.1% relative drop and 6.2% absolute drop in pregnancy-related services; Figure 2), suggesting that increased access to contraceptive services associated with postpartum Medicaid extension might contribute to decreasing the incidence of short interval pregnancies, a known risk factor for poor maternal and infant health outcomes. Moreover, Post-FFCRA MBH/SUD services utilization was 3 × the Pre-FFCRA rate (6.8 vs. 2.1%, respectively; Figure 3), thus uncovering crucial unmet needs for MBH/SUD services when women lose Medicaid coverage at 60-days postpartum (see detailed description of MBH/SUD definition in Supplementary Table 2). Additionally, outpatient utilization for preventive and problem-related services more than doubled Post-FFCRA vs. Pre-FFCRA (5.5 vs. 2.3%, and 26.4 vs. 9.8%, respectively; Figure 3). Although Post-FFCRA ED visits increased 3-fold, inpatient admissions remained unchanged (Figure 3), suggesting that ED visits might have been driven by ambulatory-care-sensitive or lower acuity conditions.

### Analysis of COVID-19 Pandemic Effects

**Figure 4:**
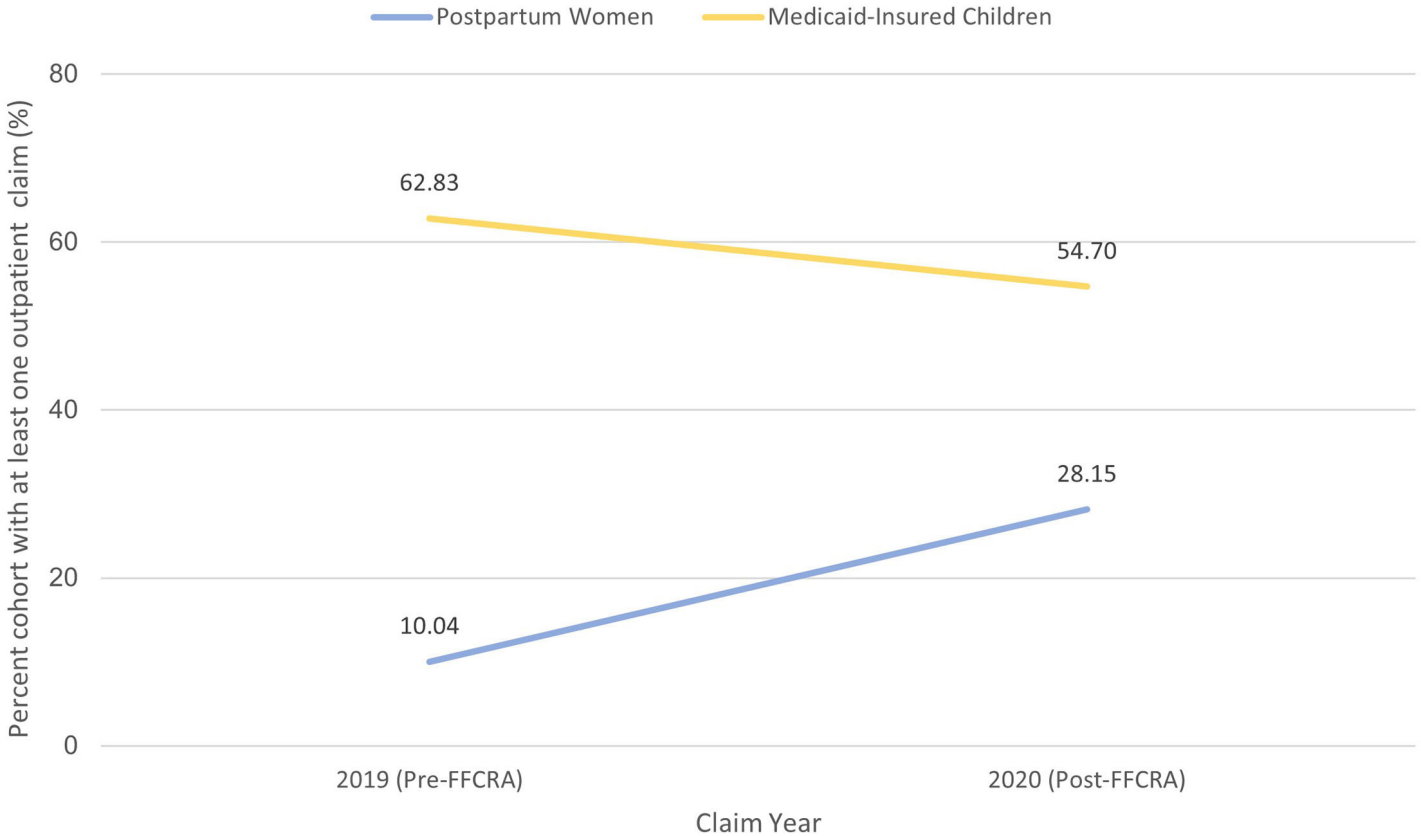
Change in outpatient services utilization among Medicaid pregnant women vs. Medicaid-insured children: isolating FFCRA impact from the COVID-19 effect.

To separate the impact of the FFCRA coverage extension from the concomitant effects of COVID-19 related disruptions on health services utilization, we compared Pre- and Post-FFCRA changes in outpatient services utilization among postpartum women vs. Medicaid-enrolled children age 2–17 (less likely to be impacted by FFCRA). Following FFCRA implementation, while outpatient utilization between 91- and 365-days postpartum almost tripled among postpartum women, overall outpatient utilization decreased by 13% among Medicaid-enrolled children (Postpartum women: Pre-FFCRA 10.0%, Post-FFCRA 28.2% vs. Children: Pre-FFCRA 62.8%, Post-FFCRA 54.7%; Figure 4). Opposite trends in outpatient services utilization among postpartum women vs. Medicaid-insured children suggest that the observed increase in utilization among postpartum women is not the sole consequence of pandemic disruptions or secular trends, and most likely the result of the FFCRA's continuous coverage requirement. These results are further supported by the finding that outpatient utilization between 90 days and 6 months postpartum were comparable between Post-FFCRA women and Pre-FFCRA women with subsequent coverage, while no utilization was observed among Pre-FFCRA women without subsequent coverage (Supplementary Figure 3).

## Discussion

Our analysis demonstrates that the FFCRA's continuous coverage requirement is associated with a sustained increase in preventive services utilization throughout the first-year postpartum. Other benefits include increased utilization of contraceptive services, decreased incidence of short interval pregnancies, and increased utilization of MBH/SUD services.

These findings are timely, as multiple state legislatures and Medicaid programs consider the necessity for and parameters of extending postpartum coverage beyond 60 days (10). The ongoing debate largely centers on the optimal duration of coverage (e.g., 6- vs. 12-months) and the breadth of services (e.g., full scope vs. limited to certain conditions) that should be provided (15). Our analysis informs the debate with empirical data supporting a broad extension of Medicaid services through the first-year postpartum, in alignment with the recommendations of ACOG and other clinician organizations as well as dozens of maternal mortality review committees (4). State-level proposals that fall short of these recommendations may fail to meet the health care needs of women in the first-year postpartum, as demonstrated by our analysis showing that about 1 in 5 women (17.9%) still seek outpatient services after 6 months postpartum as illustrated in Supplementary Figure 3. One potential consequence of unmet preventive/problem-related care—as a result of premature postpartum coverage disruptions—is an increase in uncompensated care delivered in the ED or inpatient setting. This reactive approach to care has suboptimal efficiency, incurs high societal costs, and creates a financial liability for safety net systems. Moreover, the Post-FFCRA increase in contraception utilization suggests that there may be limitations to limited-benefit Medicaid family planning programs. While some states may provide auto-enrollment into such programs at 60 days postpartum, women may not be aware of the program or the services provided, or may be hesitant to use the coverage since it is not comprehensive and may require them to change providers. Extending postpartum coverage beyond the duration of the PHE may be particularly beneficial for women living in states that have not adopted Medicaid expansion as they rely heavily on limited-benefit programs for access to health care beyond 60 days (16).

Our analysis has some limitations. First, our data is limited to a single Medicaid health plan in Texas and, though robust, is not nationally representative. Second, the observed Post-FFCRA decrease in pregnancy-related services utilization in our study could be partially explained by the well-documented decline in the nation's fertility rate associated with the COVID-19 pandemic (17). The 6% absolute decline in our study, however, is larger than the 4% decline reported in the general population by Hamilton et al. (17), suggesting that at least part of the change we report is non-pandemic-related. Third, we were unable to accurately assess the impact of the FFCRA's coverage extension across racial/ethnic groups due to incomplete documentation of race and ethnicity in Medicaid data. Fourth, we were unable to fully assess health services utilization for the Pre-FFCRA cohort due to lack of access to utilization data once Medicaid coverage was terminated after 60 days postpartum. The subgroup of women for whom data is available, including Pre-FFCRA women with subsequent coverage or women with Traditional Medicaid are not a suitable comparator for the Post-FFCRA cohort given that they meet various eligibility criteria to maintain their coverage. Overall utilization observed among Pre-FFCRA women without subsequent coverage after 60-days postpartum might be underestimated as it does not account for uncompensated or self-paid care for which data is not typically available in Medicaid claims. It remains, however, that utilization patterns in the Post-FFCRA cohort can be reasonably assumed to represent the needs of the Pre-FFCRA cohort. Lastly, we could not assess maternal and infant mortality in either cohort in the absence of vital statistics data and as this analysis was beyond the scope of this manuscript.

Future studies should be undertaken to: assess the impact of the FFCRA coverage extension across a diverse group of Medicaid health plans nationwide; carefully evaluate the impact of extended coverage on maternal and child health outcomes, including an assessment of racial/ethnic disparities; assess the impact of extending postpartum Medicaid coverage beyond the COVID-19 PHE (as states implement Section 1115 waivers and the new SPA to extend postpartum coverage); and explore the indirect effects of extended postpartum coverage on the health care safety net.

## Data Availability Statement

The data analyzed in this study is subject to the following licenses/restrictions: The datasets presented and analyzed in this study are not publicly available. This is private Medicaid and CHIP claims data for Parkland Community Health Plan members. Requests to access these datasets should be directed to GK, graham.keever@phhs.org.

## Author Contributions

YP and VC contributed to conception and design of the study. XW performed the statistical analysis. YP, XW, and EE wrote the first draft of the manuscript. EE and GK contributed to the policy interpretation of analysis. All authors contributed to critical revision of the manuscript for important intellectual content and approved the submitted version.

## Conflict of Interest

The authors declare that the research was conducted in the absence of any commercial or financial relationships that could be construed as a potential conflict of interest.

## Publisher's Note

All claims expressed in this article are solely those of the authors and do not necessarily represent those of their affiliated organizations, or those of the publisher, the editors and the reviewers. Any product that may be evaluated in this article, or claim that may be made by its manufacturer, is not guaranteed or endorsed by the publisher.
