# Supplementary material for: Extending Postpartum Medicaid Beyond 60 Days Improves Care Access and Uncovers Unmet Needs in a Texas Medicaid Health Maintenance Organization
Source: Front Public Health. 2022 May 3;10:841832. doi: 10.3389/fpubh.2022.841832 (PMC9110670; doi:10.3389/fpubh.2022.841832)
Supplement: Supplementary file 1 [file Data_Sheet_1.pdf]

Supplemental Materials

1 Additional Variables Definitions

i. Postpartum care was defined following HEDIS criteria for postpartum care, including diagnosis and procedure codes for cervical cytology screening, postpartum outpatient visits, and postpartum bundled services in the postpartum period.
ii. To analyze the reasons for outpatient utilization depicted in Figure 2, we identified all primary diagnoses for outpatient visits between 91- and 365-days postpartum using ICD-10 diagnosis codes. All ICD-10 codes for COVID-19 infection were excluded. Then, the top 30 non-COVID diagnoses associated with the highest number of claims were grouped by category and the three following categories emerged: contraception (ICD-10 Z30), pregnancy care (ICD-10 Z34 and O09), and general exam (ICD-10 Z00 and Z01).
iii. For the analysis reported in Figure 3, we applied the methodology described by Steenland et al.[14] to classify claims as preventive visits or problem-related visits i.e., visits associated with an underlying chronic condition.

2 Additional Explanation for Pre-FFCRA Cohort

Among 3,465 pre-FFCRA women, 2,807 had only one eligibility for Medicaid pregnancy coverage for the index pregnancy, which was terminated within 90 days of delivery. The remaining 658 women had subsequent coverage under new Medicaid eligibility criteria after the index pregnancy coverage was terminated:

- 298 were re-enrolled in a new Medicaid pregnancy coverage for a subsequent pregnancy,
- 125 were enrolled in Medicaid coverage for children under age 19, and,
- 235 were enrolled in Medicaid coverage for Temporary Assistance for Needy Families (TANF).
- As noted in Results and depicted in Figures S2, S3, S4 and S5, all health services utilization in pre-FFCRA cohort after 90 days postpartum occurred among women with subsequent coverage.

3 Supplementary Figures and Tables

3.1 Supplementary Figures

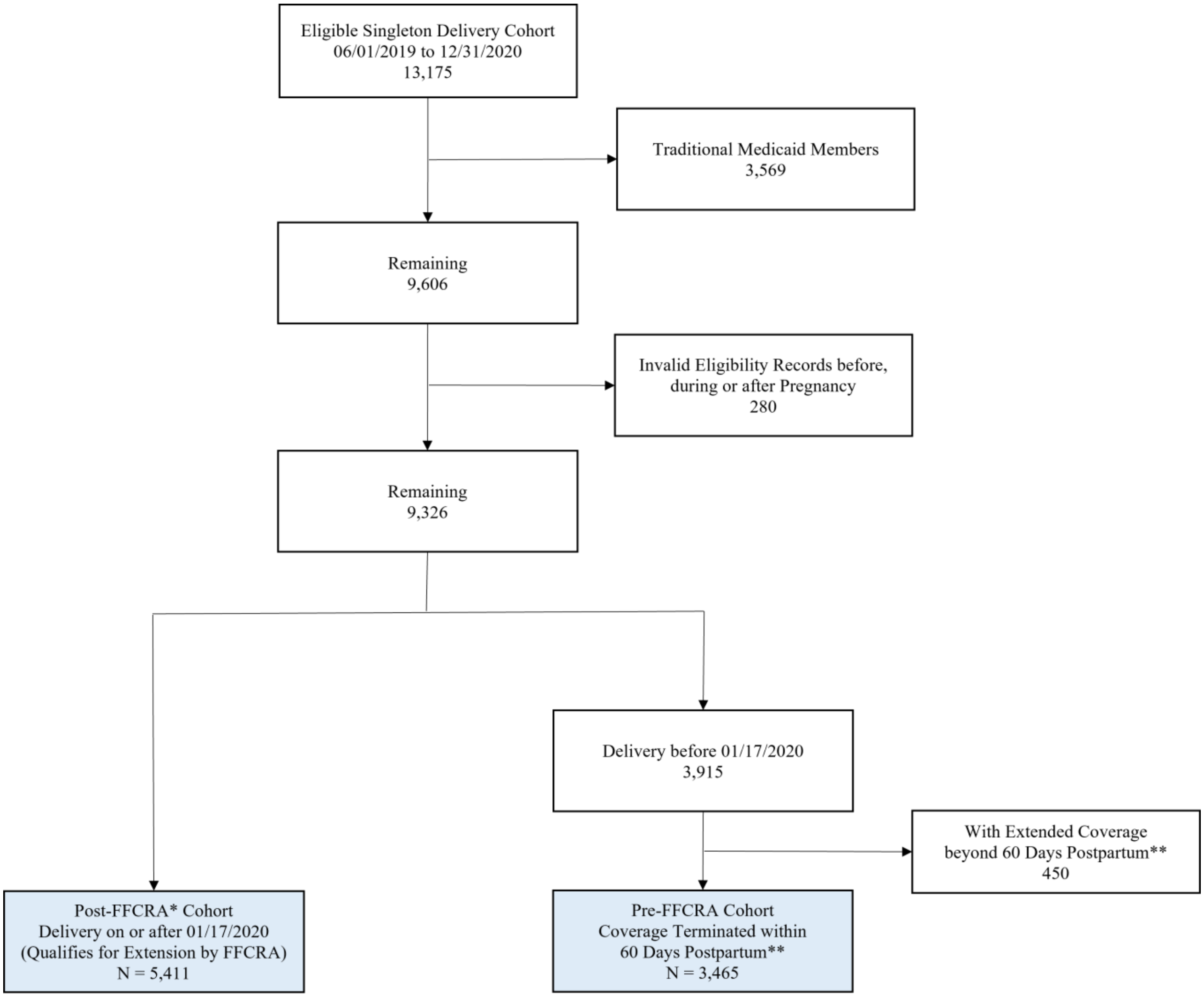

*FFCRA: The Families First Coronavirus Response Act

**60 Days Postpartum: Under federal Medicaid law, a woman who, while pregnant, is eligible for, has applied for, and has received medical assistance under the State plan, shall continue to be eligible under the plan, as though she were pregnant, for all pregnancy-related and postpartum medical assistance under the plan, through the end of the month in which the 60-day period (beginning on the last day of her pregnancy) ends (Section 1902(e)(5) of the Social Security Act).[1] This generally means that a woman will have postpartum coverage from 60-90 days postpartum.

Fig. S1. Cohort Creation

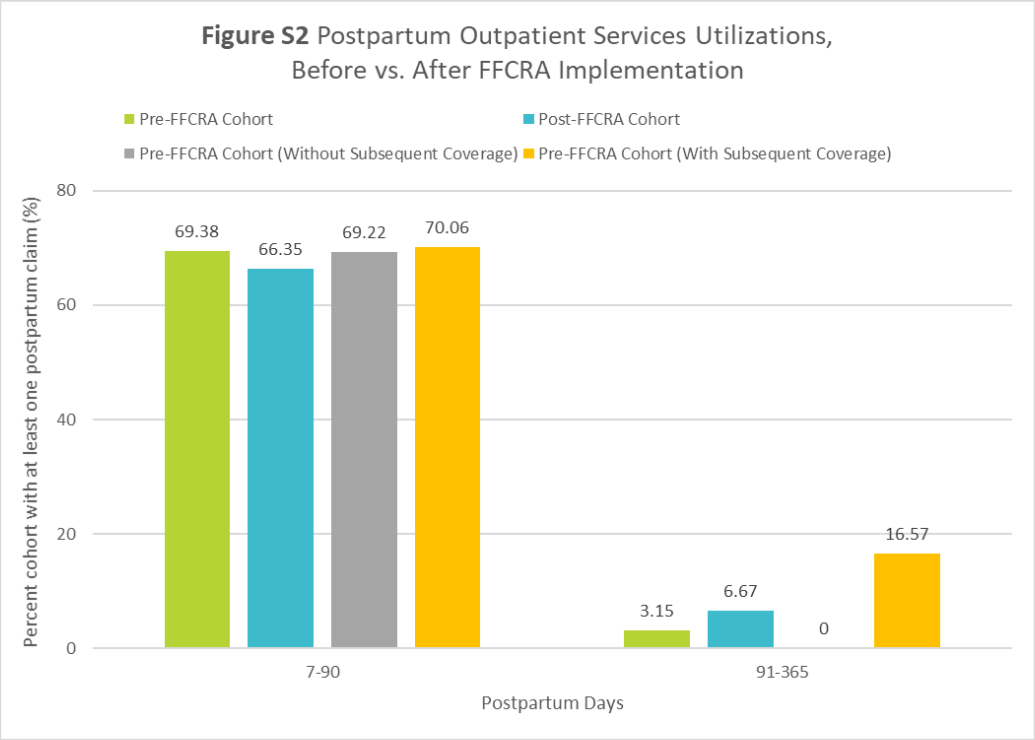

Fig. S2. Postpartum Outpatient Services Utilizations, Before vs. After FFCRA Implementation

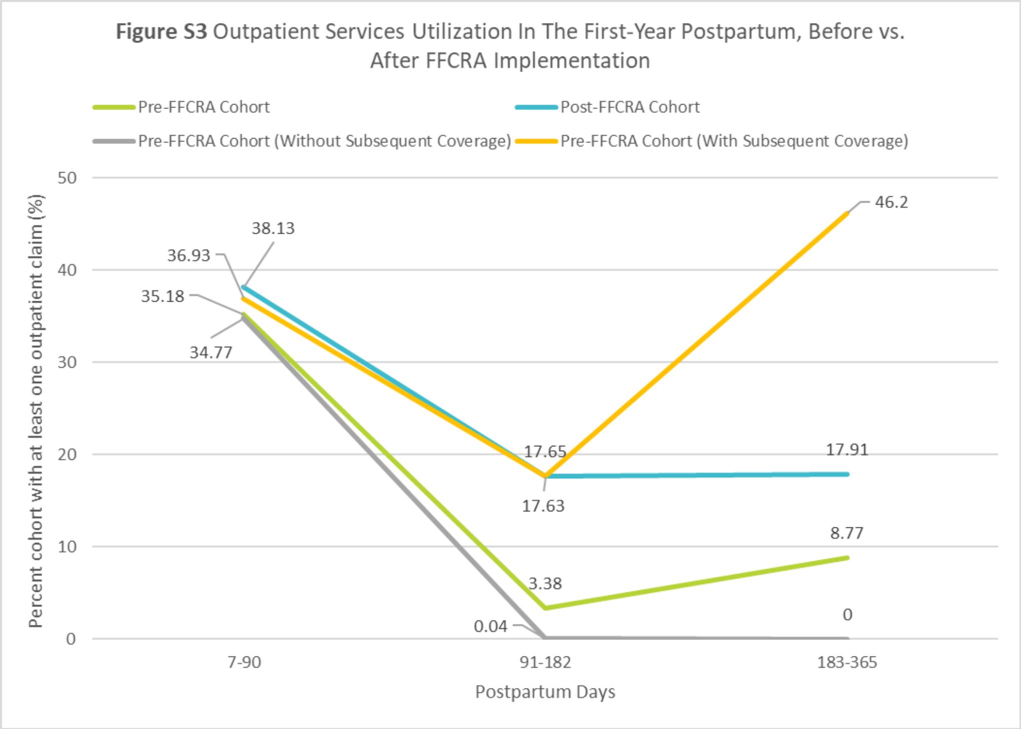

Fig. S3. Outpatient Services Utilizations In The First-Year Postpartum, Before vs. After FFCRA Implementation

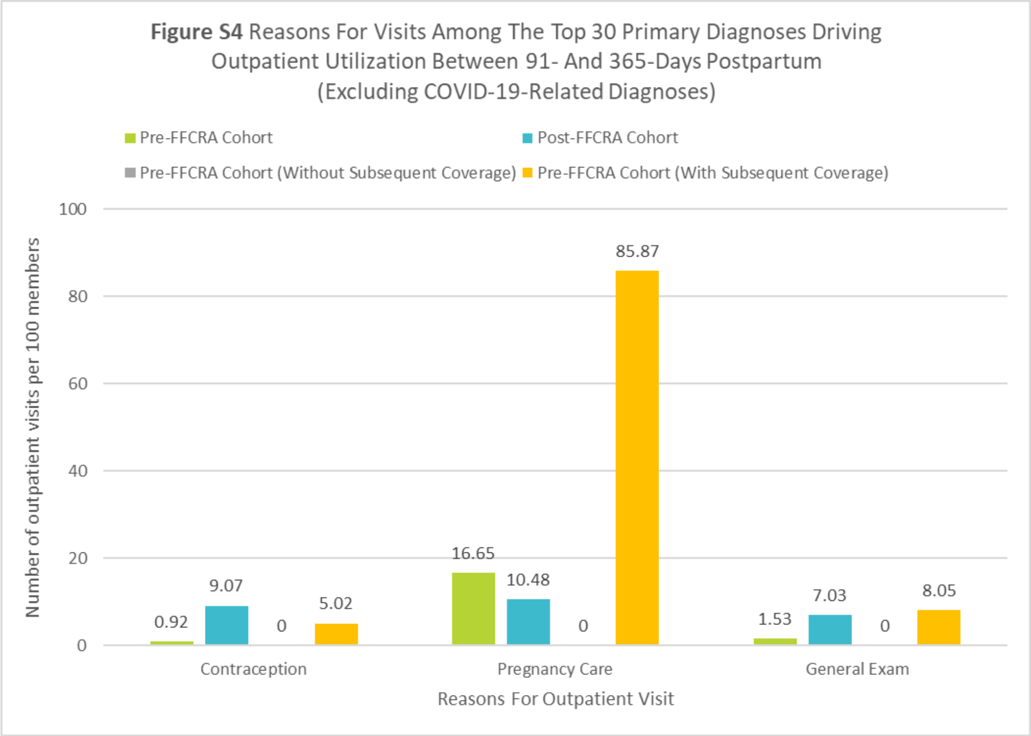

Fig. S4. Reasons For Visits Among The Top 30 Primary Diagnoses Driving Outpatient Utilization Between 91- And 365-Days Postpartum

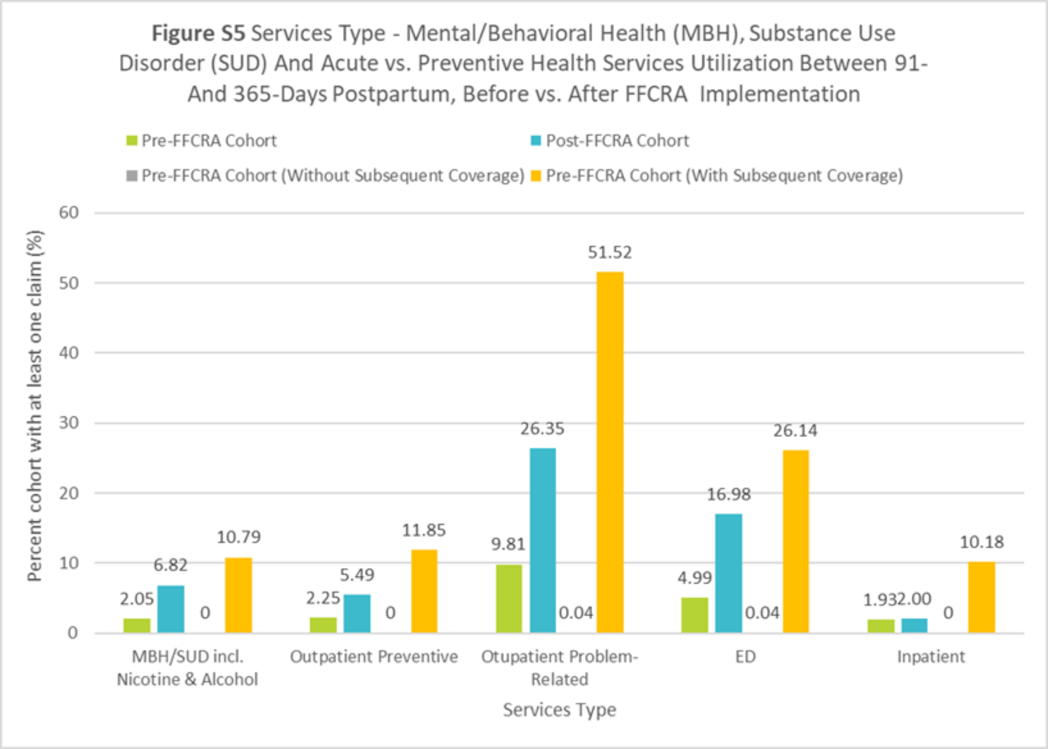

Fig. S5. Services-Type – Mental/Behavioral Health (MBH), Substance Use Disorder (SUD) and Acute vs. Preventive Health Services Utilization Between 91- And 365-Days Postpartum, Before vs. After FFCRA Implementation

3.2 Supplementary Tables

Table S1. Comparison of Baseline Cohort Characteristics

Characteristics	Pre-FFCRA cohort (N = 3,465)	Post-FFCRA cohort (N = 5,411)	*P* difference between cohorts
**Age**			
Mean ± SD, y	26.73 ± 5.52	27.05 ± 5.52	<.01
Age range, n (%)			
14-24	1,253 (36.16)	1,854 (34.26)	0.07
25-34	1,809 (52.21)	2,895 (53.50)	0.24
35-48	403 (11.63)	662 (12.23)	0.41
**Presence of chronic conditions, n (%)**			
Asthma	259 (7.47)	402 (7.43)	0.97
Hypertension	168 (4.85)	272 (5.03)	0.74
Diabetes	78 (2.25)	126 (2.33)	0.87
Mood and anxiety disorders	340 (9.81)	558 (10.31)	0.47
**Zip-level median income quartile, $**			
25th percentile	37,757	37,757	
50th percentile	46,539	46,877	
75th percentile	60,734	61,614	
**Birth outcome, n (%)**			
Preterm birth (GA* < 35 weeks)	244 (7.04%)	406 (7.5%)	0.44
Preterm birth (GA* < 37 weeks)	487 (14.05%)	797 (14.73%)	0.4

Differences in the mean characteristics in the two cohorts were tested using *t* tests.

*GA: gestational age

Table S2. Definition of Mental/Behavioral Health and Substance Use Disorder Services

Conditions	ICD-9-CM	ICD-10-CM	CPT/HCPCS/ICD-10-PCS
**Substance use disorder**	292, 304, 305.2, 305.3, 305.4, 305.5, 305.6, 305.7, 305.8, 305.9, 648.3, 648.30, 648.31, 648.32, 648.33, 648.34	F11, F12, F13, F14, F15, F16, F18, F19, O99.32, O99.320, O99.321, O99.322, O99.323, O99.324, O99.325, Z71.51	NA
**Mental and behavioral comorbidity**	290, 291, 292, 293, 294, 295, 296, 297, 298, 299, 300, 301, 302, 306, 307, 308, 309, 310, 311, 312, 313, 314, 315, 316, 317, 318, 319, V40, 648.4, 648.40, 648.41, 648.42, 648.43, 648.44	F01, F02, F03, F04, F05, F06, F07, F09, F20, F21, F22, F23, F24, F25, F28, F29, F30, F31, F32, F33, F34, F39, F40, F41, F42, F43, F44, F45, F48, F50, F51, F52, F53, F54, F55, F59, F60, F63, F64, F65, F66, F68, F69, F70, F71, F72, F73, F78, F79, F80, F81, F82, F84, F88, F89, F90, F91, F93, F94, F95, F98, F99, O99.34, O99.340, O99.341, O99.342, O99.343, O99.344, O99.345	NA
**Nicotine**	305.1, 649.0, 649.00, 649.01, 649.02, 649.03, 649.04, 989.84, V15.82	F17, Z53.01, Z71.6, Z72.0, Z87.891, T65.2XXX, O99.33, O99.330, O99.331, O99.332, O99.333, O99.334, O99.335	99406, 99407, S4990, S4991, S4995, S9453, G9276, G9458, G9497, G9902, G9906, G9908, G9909, HZ80ZZZ, HZ90ZZZ
**Alcohol**	291, 303, 571.0, 571.1, 571.2, 571.3, 305.0, 305.00, 305.01, 305.02, 305.03, 94.53, 94.61, 94.62, 94.63, 94.67, 94.68, 94.69	F10, K70, Z71.41, K85.2, K85.20, K85.21, K85.22, O99.31, O99.310, O99.311, O99.312, O99.313, O99.314, O99.315	NA
